# Field Demonstration of Heat Technology to Mitigate Heat Sinks for Drywood Termite (Blattodea: Kalotermitidae) Management

**DOI:** 10.3390/insects12121090

**Published:** 2021-12-05

**Authors:** Jia-Wei Tay, Devon James

**Affiliations:** 1 Urban Entomology Laboratory, Department of Plant and Environmental Protection Sciences, University of Hawaii at Manoa, 3050 Maile Way, Gilmore Hall 310, Honolulu, HI 96822, USA; 2 Hi-Temp Tech, LLC., 2877 Kalakaua Ave, Honolulu, HI 96815, USA; info@hitemp.tech

**Keywords:** heat treatment, termite control, termites, *Crytotermes brevis*, wood pest, heat technology, non-chemical, pest management, condominium, temperature sensor

## Abstract

**Simple Summary:**

The West Indian drywood termite, *Cryptotermes brevis* poses a significant economic threat in Hawaii, the southeast portion of continental United States, and throughout tropical and subtropical regions worldwide. Heat treatment is among the nonchemical options to manage them. A typical heat treatment may not be able to provide a complete kill of drywood termites due to the presence of difficult-to-heat areas. To mitigate this effect, studies were conducted in drywood termite-infested condominiums in Honolulu, Hawaii, where either a standard heat treatment performed by a heat remediation company or improved heat treatment methods were used. For improved treatments, heated air was directed into the drilled bases of infested cabinets for better heat penetration. Eight temperature sensors showed that sufficiently high heat was recorded at difficult-to-heat areas, including inside thick wooden cubes, for 120 min, with target temperatures of above 46 °C or 50 °C capable of killing drywood termites. A pre-treatment and a 6-month posttreatment inspection were performed to monitor termite inactivity using visual observations and by recording the numbers of spiked peaks on a termite detection device. The data showed no termite activity in improved heat treatment condominiums at 6-month posttreatment. Guidelines for the improved heat treatment are proposed.

**Abstract:**

With heat treatments to control drywood termites (Blattodea: Kalotermitidae), the presence of heat sinks causes heat to be distributed unevenly throughout the treatment areas. Drywood termites may move to galleries in heat sink areas to avoid exposure to lethal temperatures. Our studies were conducted in *Crytotermes brevis*-infested condominiums in Honolulu, Hawaii to reflect real-world condominium scenarios; either a standard heat treatment performed by a heat remediation company, or an improved heat treatment was used. For improved treatments, heated air was directed into the toe-kick voids of *C. brevis* infested cabinets to reduce heat sink effects and increase heat penetration into these difficult-to-heat areas. Eight thermistor sensors placed inside the toe-kick voids, treatment zone, embedded inside cabinets’ sidewalls, and in a wooden cube recorded target temperatures of above 46 °C or 50 °C for 120 min. Pre-treatment and follow-up inspections were performed at 6 months posttreatment to monitor termite inactivity using visual observations and by recording the numbers of spiked peaks on a microwave technology termite detection device (Termatrac). In improved treatment condominiums, significantly higher numbers of spiked peaks were recorded at pre-treatment as compared to 6 months posttreatment. Efficacious heat treatment protocols using the improved methods are proposed.

## 1. Introduction

Major drywood termite species found in Hawaii include *Cryptotermes brevis*, *Incisitermes immigrans*, and *Neotermes connexus* [1,2,3]. Among these species, the West Indian drywood termite, *C. brevis*, is the most damaging and prevalent in structures and has been recorded in Hawaii since 1884 [4]. This termite poses a significant economic threat in Hawaii and the southeast portion of the continental United States, as well as throughout tropical and subtropical regions worldwide [5,6,7,8,9,10,11]. In Hawaii, approximately USD 30 million is spent on treatment for *C. brevis* annually [12].

Wood-inhabiting insects such as drywood termites can be easily spread in infested wood products through transportation and commerce [13]. Evidence of drywood termite infestation is often the presence of fecal pellets, more commonly known as “frass,” which are usually ejected from “kick-out holes” excavated by termites in the wood surface [14]. Their cryptic nature and slow colony growth make drywood termite infestations difficult to detect until colonies are well established.

Heat is an effective means of control for pests infesting structures and commodities [15] and has been used against drywood termites, bedbugs, stored product pests, and powderpost beetles [16,17,18,19]. In general, two types of heat sources are used for heat treatment within structures: propane and electricity. Both heat source types generate and release heated air into a structure, usually with the aid of several fans to circulate the hot air throughout the target areas. During the heat treatment, temperature sensors or thermocouples are used to monitor the ambient or internal temperatures of the treatment areas. The treatment area is covered with thermal blankets or tarpaulins to contain the heated air and subsequently improve the heating efficiency. The treatment process involves heating the immediate environment of the target pest to a lethal temperature (~49 °C). Depending on several factors (e.g., the degree of infestation, structural materials, size and configuration of the treatment area, and ambient external temperature), treatment time will take from 1–2 h up to a few days to reach and maintain the target temperatures for an adequate period.

Forbes and Ebeling (1987) [20] first documented the use of high temperature of 49 °C for drywood termite management. In laboratory studies, Woodrow and Grace (1998a) [21] found that 51.3 °C was lethal to *C. brevis*, and that a wood-core temperature of 54.4 °C was sufficient to kill *C. brevis* in large timbers [22]. Scheffrahn et al. (1997b) [23] also reported that complete mortality was observed in the pseudergates of drywood termite species, including *C. brevis,* when the internal wood temperature was maintained at 54.4 °C for one hour.

One of the challenges in performing an efficacious heat treatment is the presence of structural heat sinks or difficult-to-heat areas [24,25]. These areas may be found in concrete, tile, or insulating materials contacting the target wood members. A toe-kick, sometimes known as a toe space, is the recessed area between the bottom of a cabinet and the floor. This is a common design in kitchens and bathrooms and is a potential heat sink zone. Heat sinks require higher temperatures than other areas to heat the wood sufficiently to kill termites. Furthermore, a field study by Woodrow and Grace (1998c) [26] showed that the temperature may increase slower in field applications than in laboratory studies. Depending on the degree of wood surface area exposed to heated air, a higher temperature or longer heating time may be needed to achieve the optimum internal temperature in the field.

This heat sink factor does not appear to have been thoroughly considered in developing protocols for heat treatment for drywood termite control in actual condominium units. Our research investigated the efficacy of heat treatment with the aim of eliminating or reducing heat sinks by using a novel method to direct the heated air into the toe-kick voids in condominiums infested with *C. brevis*. Using this information, guidelines for more efficacious heat treatment in condominiums are proposed.

## 2. Materials and Methods

### Study Site and Protocols

**Figure 1:**
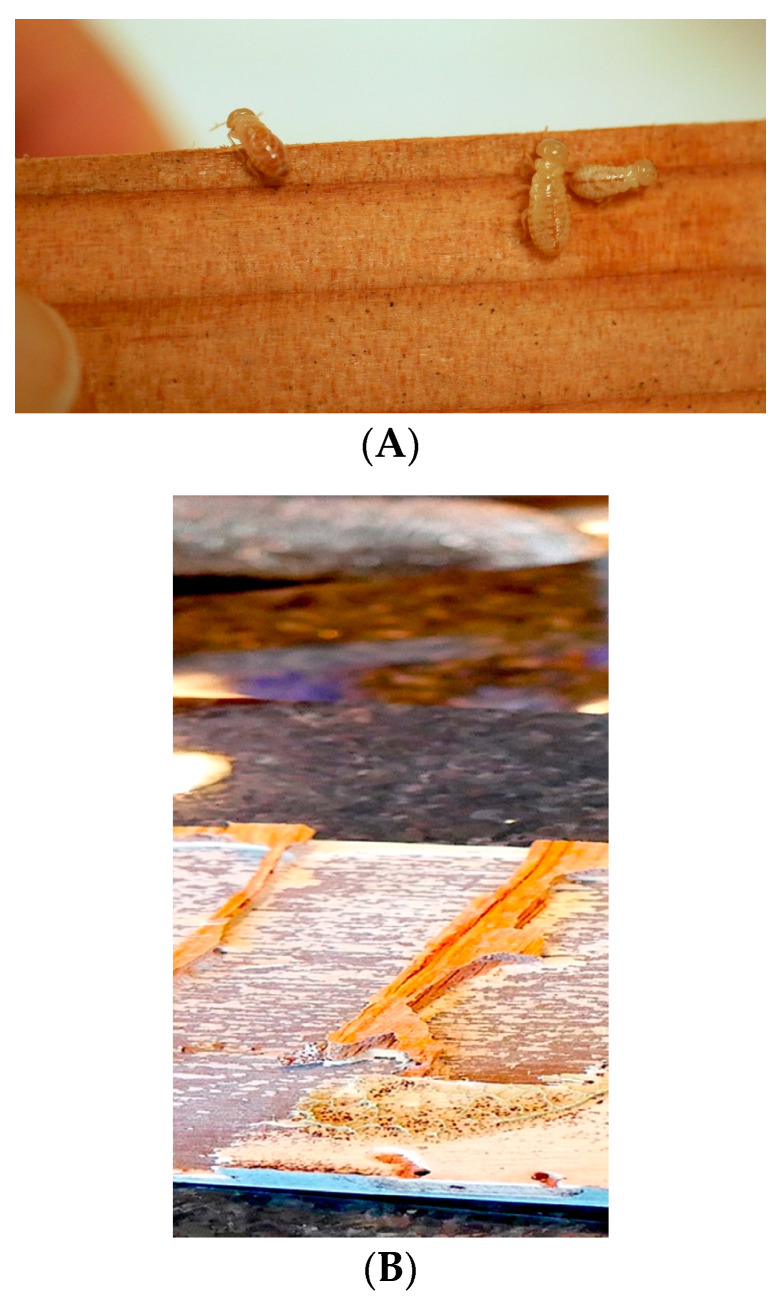
Drywood termite nested inside damaged wood were separated and transferred to another piece of undamaged wood and were kept in a transparent plastic box to facilitate general identification and photography (**A**). Part of termite damages (**B**).

The studies took place in two and three fully furnished residential condominium units in Honolulu, Hawaii, built between 2001 and 2006, for improved and standard treatments, respectively. Honolulu is located on the island of Oahu, Hawaii. This region is defined as tropical, with an average annual temperature of 20.6–27.5 °C, the ideal temperature range for termite activity. The condominium owners had noted fecal pellets in their cabinets. Upon visual inspection, *C. brevis* were found inside a few bases of the cabinets and inside the plywood countertop underlayment, and termite damage or infestation signs were found primarily at those locations (Figure 1). In each condominium, either a termite soldier or a dead soldier’s head was collected in vials with 70% ethanol and was identified in the laboratory according to an identification guide [7]. In each condominium, three 10 min inspections were carried out to confirm termite activity and the locations of infestation via observations of fluctuations in the output signal (pre-reading data not recorded) on a low-energy microwave termite detection device, commercially known as Termatrac (T3i All Sensor, Termatrac, Newport Beach, CA, USA), and through visual observation of termite fecal pellets and other termite signs. Then, the numbers of spiked peaks (i.e., the numbers of peaks with heights above average lines and above its baseline) on the output’s line graph in a 10 s period were photographed and recorded at the 10th minutes of each inspection. The device was mounted on a tripod to minimize shaking, and all residents were absent during the inspections to reduce movement around the device. All factors such as excessive wind and motion, detection of non-termite objects, movement by the person holding the device, and termite inactivity during an inspection that may generate false-positive or false-negative results on Termatrac, were considered following Taravati (2018) [27]. Heat treatments were subsequently conducted only in those parts of the condominium units (e.g., kitchens) with apparent termite activity. Hi-Temp Tech, LLC., a Hawaii-based company that provides thermal remediation services, conducted the heat treatments.

Before performing the treatments, furniture and personal items were moved away from infested areas to allow access for heat application. All electrical appliances (e.g., kettles, toasters, free standing microwaves, etc.) were removed to increase air circulation and heat penetration. Additionally, all air conditioners and air vents were covered and sealed with tape, and sink drains were taped to avoid P-trap evaporation. Fire sprinklers were insulated as necessary. These steps improve heating efficiency, help to hermetically seal the treatment zone, and reflect heat energy into the treatment areas. All cabinet doors and drawers were in open and staggered positions.

**Figure 2:**
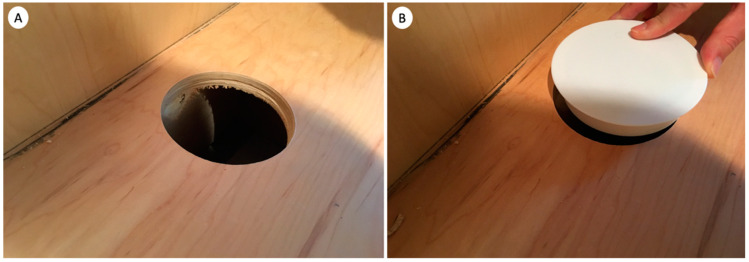
(**A**,**B**) Drilling of toe-kick voids in improved treatment condominiums (**A**). A fitted plastic cover designed to cover up the hole drilled at the bottom of cabinets after treatment for aesthetic reasons (**B**).

**Figure 3:**
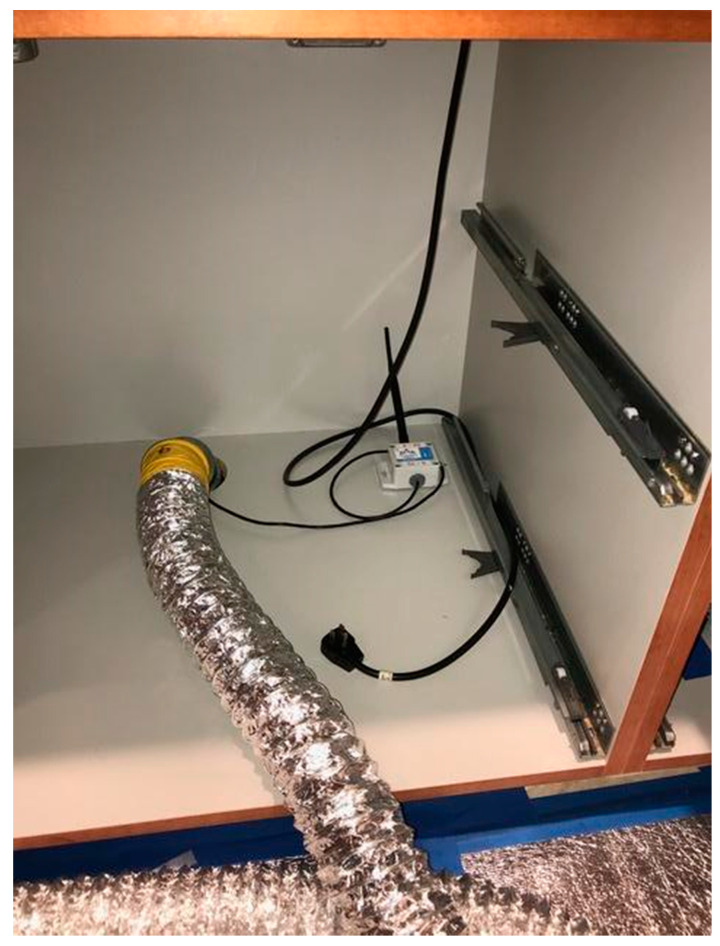
In each improved treatment condominium, probes (**right**) were placed in toe-kick voids for temperature monitoring and recording. Flexible duct pipes (**left**; with yellow cloth) were placed in toe-kick voids to direct heated air.

**Figure 4:**
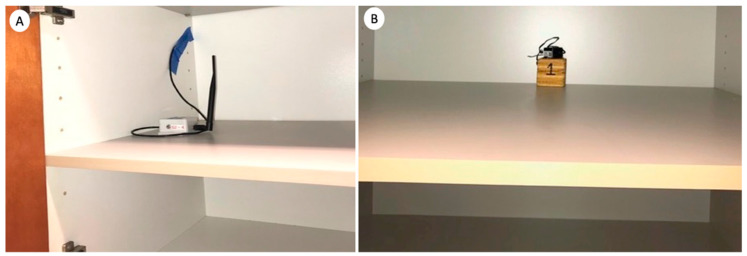
(**A**,**B**) Core temperature probes were installed by drilling to the center of the selected sidewall or side cabinet, placing the probe in the hole, and sealing the hole with duct tape (**A**). A custom-made wooden cube with an embedded temperature sensor was placed in a low-air circulation location for temperature recording (**B**).

**Figure 5:**
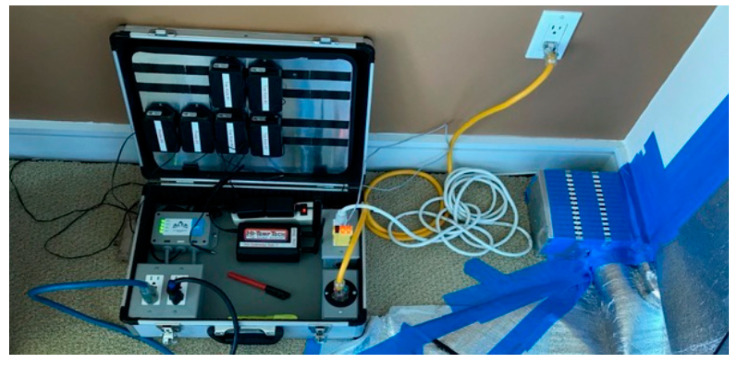
A temperature sensor control unit.

Because the *C. brevis* infestations at these condominiums were primarily associated with cabinets, the base cabinets with toe-kick voids were identified as one of the areas prone to slow rates of temperature increases. Because these areas can also be easily modified by pest management professionals, for improved treatment condominiums, four-inch-diameter holes were drilled in the back of all infested base cabinets with toe-kick voids (Figure 2). For each condominium, a total of three thermistor probes were placed deep inside the toe-kick voids next to the largest or deepest sidewall or baseboard to monitor and record temperatures to ensure that the heat thoroughly reached all areas, including these heat sink areas (Figure 3). Two ambient air temperature sensors were placed at recessed corners of the treatment zones. Two core temperature probes were installed by drilling to the center of the selected sidewall or side cabinet, placing them in the hole, and sealing the hole with duct tape (Figure 4A). Additionally, one custom-made wooden cube (9 × 9 × 9 cm) with an embedded sensor in layered woods, which simulated the thickness of the cabinet wood but may have been thicker than the actual cabinet wood to represent potential worst-case areas, was placed in a low-circulation location for temperature recording (Figure 4B). Temperatures from a total of eight wireless sensors were monitored from an online wireless portal (iMonnit, South Salt Lake, AZ, USA) in each treated condominium. A cellular gateway and control unit were set up in each treatment condominium to collect the thermistor probe and sensor readings and upload sensor data in batches at 10 min intervals to the online portal (Figure 5).

**Figure 6:**
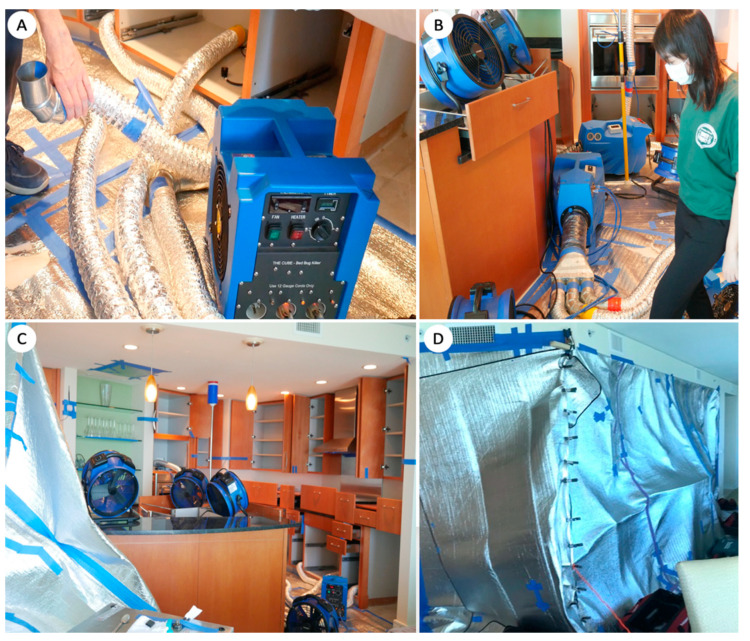
(**A**–**D**) Heaters (**A**) with distribution manifolds (**B**) and flexible duct pipes, and fans in place for heated air circulation (**C**). A treatment zone covered with a thermal blanket to contain the heated air (**D**).

Three electric heaters typically used for bed bug heat treatment, including one The Cube Bed Bug Killer (14,000 BTU’s of heat; 10–12 amps/120 V per plug; K&J Representatives LLC; Prescott, AZ, USA) and two Elite Elimination Bed Bug Heaters (20,473 BTU’s of heat; 15 amps/120 V per plug; K&J Representatives LLC; Prescott, AZ, USA), which were modified to add an attachment point with distribution manifolds, which were used to generate and direct heated air into the drilled four-inch-diameter holes in the base cabinet’s toe-kicks via three-inch-diameter flexible duct pipes (Figure 3 and Figure 6A–C). This created sufficient heated air pressure, subsequently reaching temperatures that allow the heat to transfer into all gaps, targeted spaces, and the rest of the treatment zone. The one-inch diameter difference between the duct pipes and the holes allowed the back air pressure to escape from the holes. Nine multiple angle-adjustable high-velocity fans (XPOWER X-35AR axial blower fan, XPOWER, City of Industry, CA, USA), designed to handle high temperatures without blade distortion were positioned at multiple angles. The fans were aimed to drive heated air into corners and upper cabinets and to create air currents to avoid air cavities and dead spots (Figure 6B,C). Aluminized mylar blankets were placed from floor to ceiling as necessary to create contained, hermetically sealed heat zones (Figure 6D). This improves heating efficiency and reflects energy into the treatment areas. Instead of using propane heaters, relatively safer electric heaters were used together with fans. The heating protocols were adopted from current bedbug heat treatment protocols with some modifications and improvement. In this study, once the target temperatures (46 °C or 50 °C) were reached in all voids and treatment areas, the heaters were continued for 120 min and then shut off. In the current study, heat was applied for a total of 165 min. The target temperature was achieved and maintained during the last 120 min before shutting off the heaters. The data logger stopped recording the temperatures when the heaters were shut off (e.g., at the 165 min time point) although it took additional time (data not shown) for the treatment zone to gradually return to room temperature after the heaters were shut off.

For standard treatment (as control), a heat remediation company conducted standard commercial high-temperature treatments at three residential condominiums with similar treatment zone square footage, infested cabinets and areas, and termite infestation levels. Similar heat treatment durations, temperatures, and rates of temperature increase were used and recorded. However, these treatments were performed without drilling holes in the back of base cabinets, without duct pipes to direct heated air into toe-kick voids, and without placing temperature sensors inside the toe-kick voids. For standard heat treatments, the temperature sensors were placed inside the treatment zone, embedded inside cabinets or sidewalls, or a wooden cube. The efficacy of the standard and improved heat treatments was compared at 6 months posttreatment by visual observation of termite fecal pellets and other termite signs and using Termatrac by a trained person. The signal strength, which was measured by the numbers of spiked peaks on the output’s line graph in a 10-s period, were photographed and recorded at the 10th minutes of each inspection. The data were square root transformed to meet the assumption of normality and homogeneity of variance. The numbers of spiked peaks between pre- and posttreatment for each condominium were analyzed using paired *t*-tests at *p* < 0.05 using SPSS Version 27.0 (IBM Corp., Armonk, NY, USA). The callback rates at 6 months posttreatment due to residents still noticing termite pellets or active infestations were also compared.

## 3. Results and Discussion

During the inspections prior to heat treatment, *C. brevis* were noticed within wood in the kitchens of standard and improved treatment condominiums via visual observation in the area of damaged wood, and the locations of termite infestations were further defined using a Termatrac T3i All Sensor. Hence, the results presented in the current study would be applicable to condominiums with wood cabinets, countertops, or wood furniture. Termatrac is a device that uses low-energy microwaves to detect termite motion within wood and most building materials. The Termatrac T3i All Sensor also has moisture and thermal sensors, which can detect moisture and measure surface temperatures. However, in this study, only the low-energy microwave sensor (or “radar”) was used to detect termite activity.

**Figure 7:**
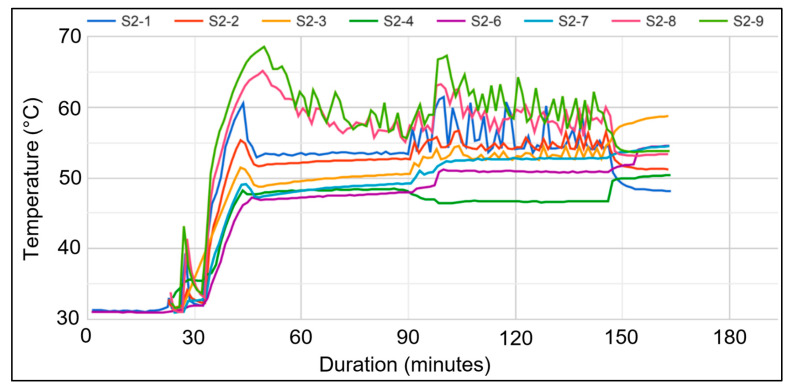
An overview of temperatures displayed on the iMonnit portal’s temperature data logger recorded by eight sensors/loggers (S2-1, S2-2, etc.) throughout the treatment period in one of the improved heat treatment condominiums. Note the fluctuating high temperatures in the top three lines, representing the toe-kick probes.

In one of the improved treatment condominiums, the temperatures from eight wireless sensors from the online wireless portal (iMonnit, South Salt Lake, AZ, USA) showed that the temperatures recorded from the toe-kick areas (the top three lines in Figure 7) fluctuated but were still able to maintain an average above 50 °C throughout the treatment period. This fluctuation occurred because the enclosed toe-kick areas can accumulate a large amount of heat, causing the heater’s internal programming to turn it on and off. The temperatures recorded from the ambient sensors (the fourth and fifth lines) and the core temperature probes inside the hole of the sidewall or side cabinet (the sixth and seventh lines) remained above 50 °C, whereas the temperatures recorded from the custom-made wooden cube with an embedded temperature sensor (the eighth line) remained steadily above 46 °C (Figure 7). These temperatures are comparable to the original suggested treatment temperature of 49 °C [28], with the exception of the embedded temperature sensor in the layered-wood cube with temperatures above 46 °C. This is because the wooden cube was made thicker than the cabinet wood to represent worst case scenarios. This indicated that if the termites were in thicker wood than that of the current study, longer durations of heat treatment would be needed. Future research using different types of buildings other than condominiums, and with different termite infestation levels is needed to build upon the current findings.

The benefits of heat treatment are the ability to treat a whole structure without using pesticides and the relatively short period for which the structure must be vacated. For example, the occupants can return immediately once the heat treatment is completed. In contrast, during fumigation, the treated structure must be aerated and must remain vacated for at least 48 h after treatment. Another advantage of heat treatment is the practicality of localized treatment, which allows it to be performed in portions of high-rise buildings, such as in apartments and condominium units, where fumigation, which must be performed in the whole structure, is not feasible. In certain circumstances, heat treatment provides greater flexibility to homeowners because they can choose whether the treatment will be applied to the entire unit or only to known areas of infestation (e.g., the kitchen, storage room, bedroom, etc.). Additionally, heat treatment has no residual toxicity, guaranteeing that the treated property is entirely safe for occupancy by humans and pets. It also poses a lower risk to applicators compared with chemical treatment [29]. No resistance or tolerance of insect pests to heat has been reported. When seeking regulatory approval, heat treatment requires less registration or authorization and thus reduces costs.

The major disadvantages of heat treatment include the difficulty or complexity of large, infested structures and the longer time required to raise the internal core of the infested wood within such structures to lethal temperatures [25]. Increasing the heating time also results in an increase in the treatment cost, the time for which an area must be vacated, and the potential for heat damage to structural items. Cost is an important factor to consider because heat treatment is generally more expensive than chemical treatment. Potential damage may also occur to heat-sensitive items, such as plastics, electrical outlet covers, and cable wiring. Sensitive products, such as aerosol cans, fire extinguishers, and adhesives, should be removed before heat treatment. Roof and wall vents should be sealed. Cleaning and vacuuming are essential before heat treatment because debris, such as food residue, may act as heat insulators.

To ensure treatment efficacy, one of the main factors that we must consider is the presence of heat sink areas [25]. Using fans to circulate heated air helps improve the treatment but does not solve the problem completely. These heat sink areas are commonly found in the wood contacting concrete floors or foundations and tile floors [30,31]. Hence, a longer heating duration is required to reach the target temperature in those areas; drywood termites tend to avoid high temperatures and may escape to these cooler zones in the wood and subsequently survive the heat treatment if the lethal temperatures are not achieved [32,33,34]. A recent laboratory study found that a temperature of approximately 49.6 °C for 2 h is required to achieve 100% termite mortality under a heat sink effect [35]. As priority should be given to monitoring and achieving lethal or targeted temperatures in worst-case areas, commercial heat treatments should consider using customized wooden cubes embedded with temperature sensors, similar as those of the current study, as an independent measurement of heat treatment effects, especially if the termites were in thicker wood.

**Figure 8:**
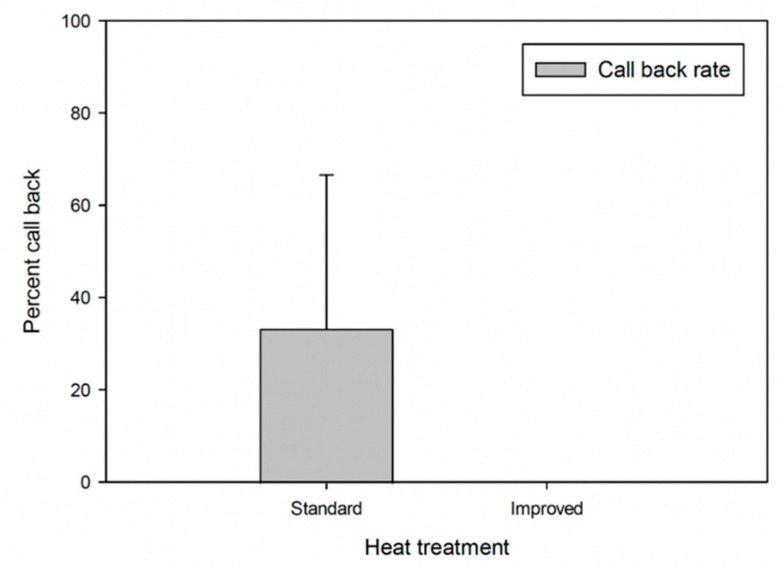
Callback (retreatment) percentages for standard and improved heat treatments (data from three standard treatment condominiums and two improved treatment condominiums).

In the current study, all improved treatment condominiums and two out of three standard treatment condominiums had no termite activity at 6 months posttreatment based on careful visual inspections and Termatrac use. Using signal strength (i.e., numbers of spiked peaks on the output’s line graph) at the 10th minutes, which were recorded and replicated three times at each condominium, the numbers of spiked peaks were statistically higher at pre-treatment as compared to 6 months posttreatment for all the improved (two out of two condominiums) and 66.67% of standard treatments condominiums (two out of three condominiums) [pre-treatment: 7.00 ± 1.15 (mean ± SEM) for standard condo 1; 9.00 ± 1.53 for standard condo 2; 9.00 ± 0.58 for improved condo 1; 9.67 ± 1.86 for improved condo 2; *t* = 11.90; 11.14; 31.09; 9.72; paired *t*-test: *p* < 0.01 in all cases] where 0.00 ± 0.00 (mean ± SEM) spiked peaks were recorded at 6 months posttreatment in these condominiums, indicating that there were no termite activity. In contrast, in one of the standard treatment condominiums, no significant difference in the numbers of spiked peaks at the 10th minutes was recorded between pre- and posttreatment (pre-treatment: 8.33 ± 2.03; posttreatment: 5.67 ± 1.76; *t* = 0.86, *p* = 0.48), and termites and their fecal pellets were found at 6 months posttreatment upon inspection. Although drilling into the toe-kick voids required more labor and a higher cost as compared to a standard heat treatment (Tay and James, unpublished data), this step increased the degree of surface exposure to heated air [26] and ensured a more effective control of the termite colonies, subsequently reduced the need for additional treatments in the future. Because only a few surviving termites were found be able to recover and develop neotenic reproductives [36], in the current study, one out of three condominiums, or a 33.33% callback (retreatment) rate occurred in condominiums that underwent the standard heat treatment, whereas a 0% callback rate occurred in condominiums that underwent the improved heat treatment after 6 months (Figure 8). Additionally, if the temperatures of all target sites including heat sink zones (e.g., toe-kick voids, etc.) can be monitored following the proposed protocols for improved treatment condominiums, either the temperature or the duration of heat treatment may possibly be reduced, shortening the period during which the structure must be vacated and reducing the treatment cost and the potential for heat damage to household items and furniture. Such careful heat treatments can provide a sustainable solution to drywood termite infestation. In addition to these efforts to improve the performance of heat treatment, a longer-term pest prevention strategy by proper building designs in new constructions (e.g., using one-piece countertop, minimizing inaccessible spaces such as spaces behind countertop and false bottom under cabinets) may reduce the prevalence of pests, including drywood termites [37].

## 4. Conclusions

Pest management professionals use several options for drywood termite treatment: local or spot treatment (i.e., pesticide liquids, foams, or dusts), fumigation, heat treatment, and others. Heat treatment is among the nonchemical options available to residents and consumers. Thus, heat treatment may be the most preferable option when residents have chemical and/or environmental concerns. Additionally, heat treatment is an attractive option for drywood termite-infested high-rise buildings, including condominium units, where fumigation is not feasible. This study was conducted in actual condominiums using actual heating protocols that are currently used in typical heat treatments, with our novel improvements. The results presented in the current study would be applicable to condominiums with wood cabinets, countertops, or wood furniture. The approaches used and the protocols developed were efficacious against *C. brevis* in this study without the use of excessive temperatures. The protocols were effective in addressing concerns regarding a standard or typical heat treatment on possible heat sink zones, and can help reduce incidences of retreatment or callbacks and possible heat damage to structural items due to the use of excessive temperatures to overcome heat sink challenges. Using the improved protocols and a thick or customized wooden cube embedded with a temperature sensor as an independent monitor and independent measurement of heat treatment effects, further investigations will be performed in detached wooden homes or other types of buildings, in addition to condominiums. Future studies can also be conducted to investigate the percentages of heat sink areas in condominiums and homes, which would be helpful when designing more efficacious heat treatments.

## Data Availability

Not applicable.
